# The Influence of Platelet-Rich Fibrin on the Healing of Bone Defects after Harvesting Bone–Patellar Tendon–Bone Grafts

**DOI:** 10.3390/medicina60010154

**Published:** 2024-01-15

**Authors:** Darko Milovanovic, Petar Vukman, Dusica Gavrilovic, Ninoslav Begovic, Lazar Stijak, Svetlana Sreckovic, Marko Kadija

**Affiliations:** 1 Clinic for Orthopedic Surgery and Traumatology, University Clinical Center of Serbia, Pasterova 2, 11000 Belgrade, Serbia; prvukman@gmail.com (P.V.); kadija.marko@gmail.com (M.K.); 2 School of Medicine, University of Belgrade, Dr Subotica 8, 11000 Belgrade, Serbia; ninobego@hotmail.com (N.B.);; 3 Institute for Oncology and Radiology of Serbia, Pasterova 14, 11000 Belgrade, Serbia; 4 Institute for Mother and Child Health Care of Serbia, Radoja Dakica 6-8, 11070 Belgrade, Serbia; 5 Center for Anesthesiology and Resuscitation, University Clinical Center of Serbia, Pasterova 2, 11000 Belgrade, Serbia

**Keywords:** ACL reconstruction, BTB graft, kneeling pain, platelet-rich fibrin

## Abstract

*Background and Objectives*: A bone–patellar tendon–bone (BTB) autograft in anterior cruciate ligament reconstruction (ACLR) is still considered the gold standard among many orthopedic surgeons, despite anterior knee pain and kneeling pain being associated with bone defects at the harvest site. Bioregenerative products could be used to treat these defects, perhaps improving both the postoperative discomfort and the overall reconstruction. *Materials and methods*: During a year-long period, 40 patients were enrolled in a pilot study and divided into a study group, in which bone defects were filled with Vivostat^®^ PRF (platelet-rich fibrin), and a standard group, in which bone defects were not filled. The main outcome was a decrease in the height and width of the bone defects, as determined by magnetic resonance imaging on the control exams during the one-year follow-up. The secondary outcomes included an evaluation of kneeling pain, measured with a visual analog scale (VAS), and an evaluation of the subjective knee scores. *Results*: The application of Vivostat^®^ PRF resulted in a more statistically significant reduction in the width of the defect compared with that of the standard group, especially at 8 and 12 months post operation (*p* < 0.05). Eight months following the surgery, the study group’s anterior knee pain intensity during kneeling was statistically considerably lower than that of the standard group (*p* < 0.05), and the statistical difference was even more obvious (*p* < 0.01) at the last follow-up. Each control examination saw a significant decrease in pain intensity in both the groups, with the values at each exam being lower than those from the prior exam (*p* < 0.01). A comparison of subjective functional test results 12 months post operation with the preoperative ones did not prove a statistically significant difference between the groups. *Conclusions*: The use of Vivostat^®^ PRF reduces kneeling pain and accelerates the narrowing of bone defects after ACLR with a BTB graft, but without confirmation of its influence on the subjective knee score.

## 1. Introduction

A bone–patellar tendon–bone graft (BTB) is, according to many authors, still considered the gold standard for anterior cruciate ligament reconstruction (ACLR). In comparison to the numerous advantages associated with a BTB graft, the flaws of its application are minor according to the existing literature and are mainly related to the donor site pathology—a patellar ligament rupture, a patellar fracture, patellar tendinitis, kneeling pain, and numbness [1]. Kneeling pain and numbness are among the most common issues that occur postoperatively among the majority of patients and persist for a long time. Kartus et al. stated that 51% of patients were unable to walk on their knees post operation with a BPTB autograft because of knee pain, while Cohen et al. stated that 42.2% of patients reported numbness 1 year after surgery [2,3]. These symptoms can be debilitating in certain activities that require strong knee support, such as wrestling, martial arts, and rhythmic gymnastics.

Some studies attempting to overcome the issue of kneeling pain with varying degrees of success can be found in the literature [4,5,6,7,8]. While one group of authors sees changing the surgical technique as a solution, which may involve a harvesting graft through two incisions, filling the bone defects with bone substitutes, and the careful reconstruction of the peritendineum, another group of authors apply regenerative medicine techniques [6,9,10,11,12,13,14].

Regenerative medicine has gained significant popularity in musculoskeletal pathology treatment over the last two decades. Platelet-rich plasma (PRP) is an autologous blood fraction that contains an increased number of platelets as well as a broad spectrum of cytokines, such as platelet-derived growth factor (PDGF), vascular endothelial growth factor (VEGF), transforming growth factor beta-1 (TGF-B1), fibroblast growth factor (FGF), and insulin-like growth factor-1 (IGF-1) [15]. Together, they have great potential for the regeneration of tissues which have a poor healing capacity. PRP could enhance the healing action via improved adhesion, recruitment, proliferation, migration, and the differentiation of stromal cells, and participate in tissue remodeling, matrix production, and chondrogenic differentiation [16].

Due to the development of regenerative medicine, new therapeutic modalities have emerged, including platelet-rich fibrin (PRF), which contributes to the additional acceleration of musculoskeletal tissue recovery. This preparation technology is based on increasing the concentration of platelets, fibrin, growth factors, and cytokines that promote tissue regeneration by stimulating mesenchymal cells and macrophages together with osteoblasts [17]. Skarpas G. found that the usage of PRF in soft tissue, bone, and cartilage lesions results in better functional recovery and less intense pain, which could last up to 12 months post operation [18]. PRF is formed by centrifuging autologous blood using the Vivostat^®^ System (Vivostat A/S, Lillerod, Denmark). This process has a dual benefit, considering that compared with unprocessed blood, the obtained product has a 7-fold higher platelet concentration and a 7–10-fold higher fibrin concentration, as well as a significantly reduced concentration of matrix metalloproteinase-9 (MMP-9), which is an enzyme that can inhibit tissue healing [19]. Thanks to its composition, PRF is a cause of significant regeneration [20]. It has been proven that PRF increases the expression of the psoriasin gene (S100 calcium-binding protein A7) in in vitro conditions, which is responsible for angiogenesis, wound healing, and immunomodulation [21]. Considering all the above mentioned facts, in addition to the literature data, it can be concluded that PRF has been frequently used in combination with bone graft materials to reduce the healing time and promote bone regeneration [13].

The aims of this research were to examine the regenerative capacity of Vivostat^®^ PRF as well as its impact on reducing pain intensity through subjective and kneeling tests.

## 2. Materials and Methods

### 2.1. Study Background and Objective Limitations

The first idea, which was to organize this study at the Clinic for Orthopedic Surgery and Traumatology, University Clinical Center of Serbia, as a prospective randomized single-blind study to investigate the efficacy of the Vivostat preparation in a cohort of male patients with ACLR actively engaged in sports, was abandoned. Due the problems and limitations caused by the COVID-19 pandemic, this research was set up and approved as a pilot cohort study by the local Ethics Committee and conducted from March 2021 to March 2022, including a sample size of 59 patients [22]. Including the planned follow-up period of 12 months after surgery, this study was completed in March 2023.

The inclusion criteria were as follows: male patients who were active athletes, aged 17–45, with an isolated injury to the anterior cruciate ligament of the knee, and without clinical and radiographic signs of osteoarthritis. All patients signed an informed consent form to participate in the study and were operated on by the same surgeon.

The research design involved two groups: Vivostat (donor site defect filled with Vivostat^®^ PRF, Vivostat A/S, Lillerod, Denmark) and a standard group (donor site defect not filled with any material).

**Scheme 1:**
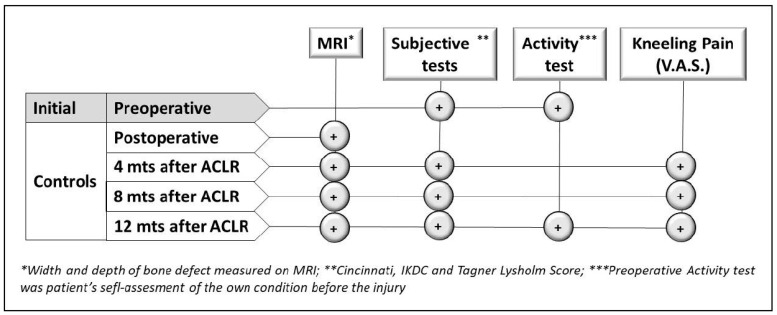
Dynamics of measurements and controls.

The follow-up included a clinical examination, tests (subjective, activities, and kneeling pain), and magnetic resonance (MR) findings at 4, 8, and 12 months after arthroscopic ACLR, as shown in Scheme 1.

The exclusion criteria referred to the patients who previously underwent surgical procedures on the injured or contralateral knee; those with intraoperatively verified lesions of the meniscus or articular cartilage; people with postoperative complications (infection, arthrofibrosis and deep vein thrombosis); noncompliant patients (failure to attend follow-up examinations or being late for longer than 15 days); and patients whose exclusion was caused by technical problems (MRI out of order; some patients left the country).

**Figure 1:**
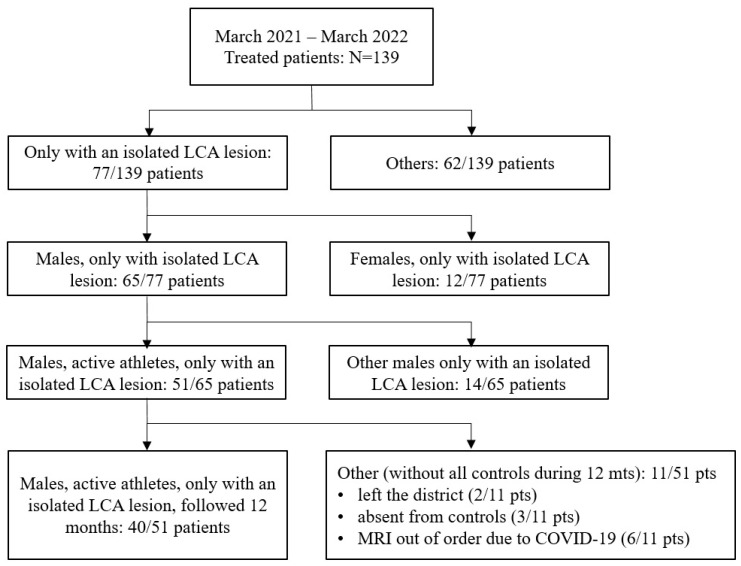
Flowchart of patient selection.

Due to the reduced number of patients available during the COVID-19 pandemic, the final analysis was conducted on 40/139 patients (20 participants per group) with ACLR who met all the criteria for participation (Figure 1), similar to in other studies [12,17,23,24,25].

Using the results of our study, which included a mean difference of 1.07 points for the VAS kneeling pain score, an SD difference of 1.27, an alpha value of 0.05, and a sample size of 40 patients, a two-tailed test confirmed that our study had a power of 83.77%.

### 2.2. Vivostat^®^ PRF Preparation

The Vivostat^®^ PRF system (Vivostat A/S, Lillerod, Denmark) is a product kept in sterile packaging and used for blood preparation in order to obtain platelet-rich fibrin. The process begins with venipuncture and collecting about 120 mL of autologous blood from the donor 15 min before the induction of anesthesia. The blood is directly collected into a single-use sterile bottle, where an automated process concentrates fibrin and platelets. This preparation takes up to 25 min and results in 5–6 mL of the final product. The automated process takes place in the preoperative holding area, and after centrifugation, it is introduced into the operating room. A cassette with the final product is put into the Vivostat^®^ applicator, where the solution is buffered. A sterile system directly connected to the applicator unit has the option to spray the solution. Due to the donor site being filled as per the conditions of our study, the system was connected to the spray applicator shaped like a low-pressure spray pen, enabling precise solution application. The applicator unit was activated with a foot pedal or by pressing the spray applicator button. The application process itself takes 2–3 min, after which immediate polymerization and the formation of a bioactive fibrin matrix occur. From that moment, the slow release of growth factors begins, lasting 7–10 days.

### 2.3. Surgical Technique and Postoperative Treatment

The surgery was performed under general endotracheal anesthesia in surgical hemostasis using a tourniquet device. Access to the patellar ligament was achieved through a longitudinal incision in its medial edge projection, with an average length of 6 cm. After soft tissue dissection, the peritendineum was opened with a longitudinal incision, revealing the patellar ligament in its entirety. The BTB graft was harvested along the total length of the ligament, with a width of 10 mm and bone blocks measuring 20–25 mm in length. The reconstruction of the anterior cruciate ligament was performed arthroscopically, with the positioning of bone tunnel openings at the anatomical attachments of the ligament, and the graft secured with osteoconductive interference screws (BioComposite Interference Screw, Arthrex, Naples, United States).

**Figure 2:**
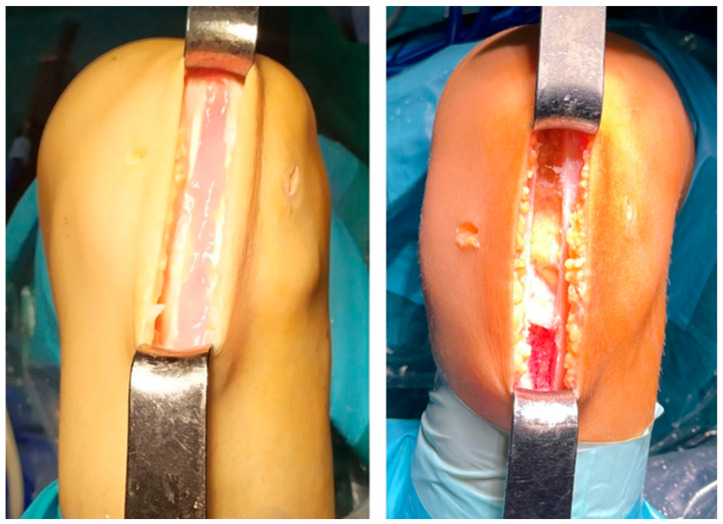
Vivostat group (**left**) vs. standard group (**right**).

Donor site bone defects of participants in the Vivostat group were filled with Vivostat^®^ PRF (Figure 2—left), while the bone defects of participants in the standard group were not filled with any material (Figure 2—right).

The peritendineum edges were reconstructed in both groups using absorbable Polysorb^TM^ 3-0 sutures, while the subcutaneous tissue and skin were sutured in the usual manner. A surgical drain was placed through the anterolateral portal and remained in place for up to 24 h after the surgery, while no drain was placed in the donor region. Postoperative analgesia was achieved with a multimodal concept, combining a peripheral nerve block (adductor canal block) and parenteral analgesics. All patients received a COX-2 inhibitor on the first postoperative day and then during the next 14 days. Early rehabilitation treatment started from the first postoperative day, initially with bed exercises in order to achieve a full range of motion, followed by walking with crutches and gradual weight-bearing on the operated leg. All participants underwent an identical rehabilitation protocol.

### 2.4. Magnetic Resonance Imaging (MRI)

**Figure 3:**
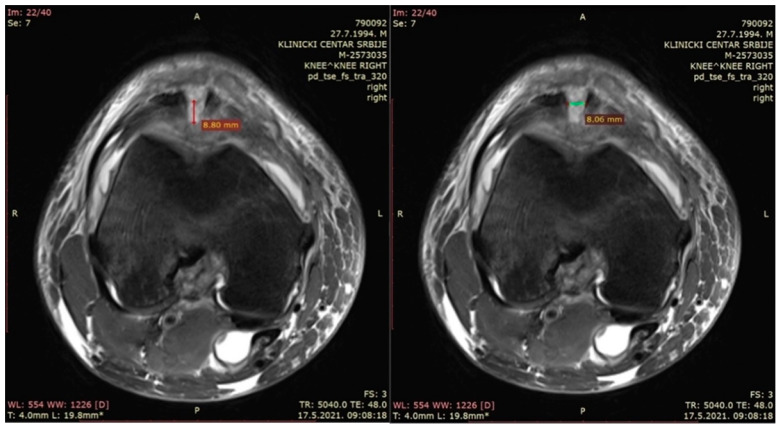
Patellar bone defect. Depth (**left**) vs. width of the defect (**right**).

Postoperative magnetic resonance findings were obtained immediately after the surgery, as well as during the follow-up examinations (Scheme 1). MRI analysis was conducted in the Center for Magnetic Resonance of the University Clinical Center of Serbia (Siemens Healthcare GmbH, Magnetom Skyra 3T, Erlangen, Germany). During study initiation, an MRI examination protocol was defined. The obtained findings were analyzed with RadiAnt DICOM Viewer 2023.1 (64-bit) software. Two variables were defined when analyzing the obtained DICOM cross-sections, whose sample values were measured immediately after the surgery and during all the follow-ups:

− The depth of the patellar bone defect (mm)—a variable used to assess the depth of the bone defect from the first section caudal to the top of the defect in a transverse plane in the t2_tse_sag sequence at the center of the defect, as defined using the sagittal plane (Figure 3—left).
− The width of the patellar bone defect (mm)—a variable used to assess the width of the bone defect from the first section caudal to the top of the defect in a transverse plane in the pd_tse_fs_tra sequence (Figure 3—right).

### 2.5. Kneeling Test

The presence of pain in the front of the knee was assessed at the follow-up examinations. Initially, the patients were asked “Do you have pain in the front region of the knee?”, to which they responded with yes or no. Further assessment of the patellar pain was performed with the kneeling test. The test was conducted in the 4th month after surgery. The test was very short, and it was performed on a firm surface, requiring the patient to kneel on the surface for 10 s without moving in order to adapt to the surface and evenly distribute their weight between both knees. If the subject was unable to tolerate the pain in this position, the test was terminated, and the highest degree of pain was noted. If kneeling in this position was feasible, the test was continued by having the patient take three steps alternately with each knee on the surface. The results of the test can vary from an inability to kneel, as already mentioned, to an ability to kneel but an inability to take any steps, pain during kneeling, discomfort during kneeling, and kneeling with no issues. If pain was present when performing the test, its intensity was assessed using the visual analog scale (VAS), expressed in absolute numbers with a range of 0–10. In our study, the test was conducted at all the follow-up examinations, starting on the 4th month after surgery, and the initial condition in both groups was monitored, as well as the dynamics of the symptoms since the initial examination throughout all the follow-ups until the final examination.

### 2.6. Questionnaires and Evaluation of Donor Site Morbidity

The assessment of quality of life and evaluation of donor site morbidity was conducted using:

- Knee functional questionnaires (Modified Cincinnati Rating System Questionnaire, Tegner Activity Level Scale, IKDC Subjective Knee Evaluation Form, and Tegner Lysholm Knee Scoring Scale).
- Subjective perception of kneeling pain (VAS: 0–10).

The questionnaires were filled out and the subjective perception/sensation of pain was measured during the kneeling test at all the follow-up examinations (Scheme 1).

### 2.7. Statistical Analysis

For normal distribution data testing, the Kolmogorov–Smirnov and Shapiro–Wilk tests were used. The data were summarized using descriptive statistical methods (frequencies, percentages, mean, median, standard deviation (SD), and range). The statistical significance level was set at *p* < 0.05, and Bonferroni correction was used for multiple testing of the same dataset. The Wilcoxon rank-sum and Pearson chi-squared tests were used to compare the characteristics of the patients, diseases, treatments, and outcomes among the investigated groups. For testing the differences between measurements and controls, Friedman and Wilcoxon signed-rank tests were used. In order to avoid bias, analysis included an examination of the correlation (Spearman’s rank rho) between the bone defect size and kneeling pain (VAS). The statistical analysis was conducted with the program R (version 4.3.1 (2023-06-16 ucrt)—“Beagle Scouts”; Copyright © 2023 The R Foundation for Statistical Computing; Platform: x86_64-w64-mingw32/x64 (64-bit)) (available at: www.r-project.org; downloaded: 21 August 2023).

## 3. Results

Our study included 40 patients; general, disease, and treatment characteristics for both the groups (Vivostat and standard) are presented in Table 1.

During the follow-up period, no complications, such as postoperative infections, inflammation, or coagulation disorders, were noted. In order to objectively assess the reduction in the dimensions of the patellar bone defect (depth and width, both in mm), the magnetic resonance findings (in the fourth, eighth, and twelfth months after surgery; Scheme 1) are shown in Table 2 and Table 3.

Analysis of the depth of patellar bone defects confirmed no statistically significant differences between the study groups in all the measurements (Table 2). However, comparisons between all the controls and in both the groups confirmed a statistically significant reduction in the depth of patellar bone defects (Table 2).

Bone defect width analysis confirmed statistically significant lower values in the Vivostat group in the eighth and twelfth months after surgery compared with the standard group (Table 3). Similar to the previous dimensions of bone defects, comparisons between all the measurements (preoperatively in the fourth, eighth, and twelfth months after surgery) confirmed a statistically significant reduction in the patellar bone defect width for all the controls and both the groups (Table 3).

The results of the subjective test analysis (Modified Cincinatti, Tegner Activity, IKDC and Tegner Lysholm score—initial/preoperative/and values 12 months after surgery; Scheme 1) confirmed no statistically significant difference between the Vivostat and standard groups (Table 4). However, statistically significantly higher Modified Cincinatti, IKDC and Tegner Lysholm scores were recorded in the last measurement, overall, and within the two study groups, where lower activity scores were also noted, but without statistical significance (Table 4).

In order to avoid bias in the analysis of pain during the kneeling test, the correlations between the dimensions of the bone defect (depth and width, in mm) and the intensity of pain (VAS) during the kneeling test for all the patients are presented in Table 5. Due to very poor correlations, the results of a similar analysis of the Vivostat and standard groups are not shown.

Pain intensity analysis (VAS) during the kneeling test confirmed significantly lower values in the Vivostat group at 8 and 12 months after surgery compared with those of the standard group (Table 6). Comparisons of all measurements (in the fourth, eighth, and twelfth months) showed a significant decrease in pain intensity in the last two controls (eight and twelve months after surgery) overall and in the Vivostat group (Table 6). In the standard group, statistically significantly less pain was measured at the last follow-up control (twelve months after surgery; Table 6) compared with the earlier pain values (four and eight months after surgery).

**Figure 4:**
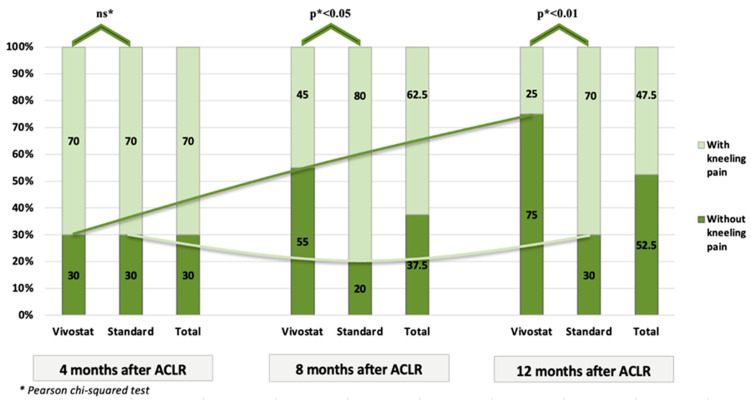
Kneeling pain (yes/no) during control examination in Vivostat, standard, and the whole sample group.

In addition to pain intensity (VAS) during the kneeling test, the presence/absence of pain during the control period (four, eight, and twelve months after surgery) was analyzed, and the obtained results confirmed significantly more pain-free patients in the Vivostat group during the last two controls (eight and twelve months) compared with those in the standard group (Figure 4).

## 4. Discussion

The beginning of the 20th century was marked by the significant development of regenerative medicine, which, over time, proved to be an important asset in the treatment of certain orthopedic pathologies [14]. Initial studies assessed the impact of bioregenerative products on improving postoperative ACLR results. Some authors researched the role of regenerative medicine in the accelerated biological integration of soft tissues in bone tunnels, as well as its significance in the graft maturation process and prospective ligamentization [26,27]. At the start of the 2010s, a few publications were published indicating the role of regenerative medicine in reducing the postoperative symptoms, such as decreased pain intensity or the acceleration of sensory recovery in the donor site region, after harvesting a BTB graft.

The three most commonly used grafts for ACLR treatment for active athletes are performed on the BTB, hamstring tendons, and quadriceps tendons [28,29]. Authors of other studies tend to favor a certain type of graft and criticize other methods. In most cases, conclusions are drawn by monitoring the re-rupture rate and donor site pathology. The literature demonstrates that the hamstring tendon might exhibit a slightly higher re-rupture rate when compared with that of the BTB, but the BTB causes more kneeling pain in the short- and mid-term follow-ups [30]. The reason for the occurrence of postoperative symptoms lies in the donor site defect formed after BTB graft harvesting [4]. Despite all of the prior available studies, it can be concluded that in the arthroscopic reconstruction of the anterior cruciate ligament, the gold standard is the use of a BTB graft, with all of its advantages and disadvantages.

The results of the study conducted at the Clinic for Orthopedic Surgery and Traumatology of the University Clinical Center of Serbia demonstrated homogeneity among the study groups by analyzing the basic demographic features and injury characteristics (Table 2). Analyzing the obtained values from the international functional questionnaires (Modified Cincinatti, IKDC and Tegner Lysholm score), statistically significantly higher values were proved 12 months after the surgery compared with the initial preoperative findings (*p* < 0.01) both overall and in the separate groups. These findings suggest that ACLR leads to both subjective and functional improvements in all surgically treated patients, regardless of whether the donor site defect is filled with Vivostat^®^ PRF or not. Comparing the values of the obtained tests at the initial and follow-up examinations among the study groups did not prove any statistically significant differences, which indicates that filling the patellar defect with Vivostat^®^ PRF does not cause improved functional recovery.

The Tegner Activity scores in the overall sample before the injury were relatively high in our study, considering that we were focused on the problems among a cohort of active athletes. Comparing the results among the groups, no statistically significant differences were found preoperatively or 12 months postoperatively. Analyzing the Tegner Activity score values before the injury and one year after the surgery using the Wilcoxon signed-rank test, no statistically significant differences were found in either the overall sample or the separate study groups (*p* > 0.05; Table 3). The obtained results indicate that the treatment method is not crucial for a better activity score result one year after the surgery (Table 3). The data we obtained are comparable to those in other studies; in most of the published studies, a return to the pre-injury activity levels was noted one year post operation [31]. The studies rarely show a significant improvement in the postoperative Tegner Activity score compared with pre-injury levels, and such findings are usually found in recreational athletes [32]. In such situations, initially low postoperative score values show a significant increase, most likely due to the patient’s will and motivation to return to sport activities after surgery.

The MRI findings obtained immediately after surgery, as well as at each follow-up examination, are crucial for objectifying the assessment of bone defect filling. In our study, we measured the depth and width of the bone defects on all four MRI scans of each individual patient and at a predefined position to ensure data coherence. The measurement of the bone defect depths at the initial, immediate, and postoperative examinations, as well as on follow-up MRI scans, did not show statistically significant differences among the groups. By observing the differences among the measurements in the overall sample, a statistically significant reduction in the bone defect depth values over time was verified through the Friedman test (*p* < 0.01) on all the follow-up measurements. This finding directly confirms that bone defect filling occurred in all the patients over time, which was also confirmed through the Wilcoxon test with Bonferroni correction (*p* < 0.0083; Table 4). Analyzing the Vivostat and standard groups separately, an identical situation was observed as in the overall sample; the depths of the defects decreased over time and showed more statistical significance at each follow-up examination compared with the previous one. This finding confirms the recovery of the bone defect over time, whether the patients were treated using Vivostat^®^ PRF or routinely; however, no statistical differences between the groups were verified regarding the measured depths of the bone defects (Table 4).

The measured values of bone defect widths did not show statistically significant differences at the initial postoperative MRI examinations among the study groups, which also points to coherence among the groups (Table 5). The first follow-up examination, which took place four months after the surgical treatment, also did not show statistically significant differences among the groups, but at the subsequent follow-up examination eight months after surgery, statistical significance was noted (*p* < 0.05), as well as on the final follow-up examination twelve months after the surgery in both cases (*p* < 0.01; Table 5). This implies that the filling of the bone defects occurred more rapidly in the Vivostat group. By observing the differences between measurements using the Friedman test, both overall and in each group, the presence of statistically significant differences was proved in all the measurements (*p* < 0.01). The Wilcoxon test with Bonferroni correction showed a decrease in the bone defect widths, with more statistically significant differences in all the follow-up examinations compared with those of the previous and initial examinations (*p* < 0.0083) both overall and in each group (Table 4).

The measurements of the dimensions of the bone defects conducted in our study correlate to published studies regarding the regeneration and repair of bone defects over time [11,24,33]. Walters and colleagues also assessed, using MRI diagnostics, the reductions in bone and ligamentous defects. Their research relied on assessing defect filling at the time of the patient’s return to sports, which, in their research, occurred between the sixth and ninth months. Their study found no statistical differences between the PRP group and the control group [11]. Additionally, Cervelin et al. observed how PRP affected the filling of donor site defects. The review was based on the variations in donor site morphology between the two groups. Through their investigation, they demonstrated that one year following surgery, defects were filled in 85% of patients in the PRP group, whereas 60% of patients in the group where the defect was not filled with PRP experienced satisfactory outcomes [23]. Therefore, considering the results of our study, as well as those of the other authors who have engaged in similar topics, we can confidently state that the filling of bone defects will undoubtedly occur over time. The only question that remains is whether the application of any of the bioregenerative products will lead to a defect filling faster.

As already mentioned, anterior knee pain is a very common problem in patients who have undergone LCA reconstruction, where the middle third of the patellar ligament was used as a graft [29]. In our group, anterior knee pain was noted in 70% of all the patients at the first follow-up examination (Figure 4). The average pain value measured with the VAS during the kneeling test was 1.7 ± 1.49 in the overall sample, while in the Vivostat group, the value was 1.7 ± 1.53, and it was 1.7 ± 1.49 in the standard group (Table 5). The analysis of the results obtained with the Wilcoxon test showed no statistically significant differences. At the next follow-up examination, eight months after the surgery, a reduction in pain intensity was noted both in the overall sample and in the Vivostat and standard groups: 0.75 ± 1.07 vs. 1.5 ± 1.1. Analysis of the differences between the study groups demonstrated a statistically significant difference in favor of the Vivostat group (*p* < 0.05). Further analysis of the consequent follow-up examination, 12 months after the surgery, still showed a decrease in pain intensity in both the Vivostat and standard groups, 0.35 ± 0.75 vs. 0.9 ± 0.79, but this time, with an even more significant statistical difference between the groups in favor of the Vivostat group (*p* < 0.01; Table 5). All of this indicates that in the group treated with Vivostat^®^ PRF, the number of patients without complaints was higher at each follow-up examination than the patients treated with the standard surgical technique (Figure 4). The values within each group, as well as the overall sample, decreased over time; therefore, we also assessed the differences within the groups using the Wilcoxon signed-rank test for pairs and repeated measurements, which showed significant decreases in pain intensity in all the measurements compared with all of the previous ones both overall and in the Vivostat group. However, the standard group test data are somewhat different. More significant differences were noted only at the final follow-up examination, twelve months after surgery, compared with those at the follow-up examinations four and eight months after surgery, while the follow-ups at four and eight months after surgery revealed no statistically significant differences. These data definitively show that the use of Vivostat^®^ PRF can more potently reduce the pain intensity, which was clinically noted significantly earlier than the standard group experienced.

We compared the results obtained with those of other authors. Kovindha et al. also assessed kneeling pain intensity using the VAS among patients who had a patellar bone defect which was not filled in their study [29]. The average value of pain intensity was 2.0 ± 2.5 six months after surgery, which is slightly higher than the average values in the standard group in our study. The shortcoming of the study published by Kovindha is the lack of a group in which the defect was filled with some orthobiological products in order to make the results comparable. However, their results are in accordance with the data we obtained in the standard group, which is sufficient for us to compare them with the patients from our study who had a defect filled with Vivostat. Therefore, it can clearly be concluded from our results that Vivostat leads to an increased reduction in pain intensity over time compared with that of patients treated with the standard treatment, as well as the faster disappearance of pain in the anterior knee region.

Cervelin et al. assessed kneeling pain intensity using the VAS 12 months after surgery and filling of the bone–ligament defect with platelet-rich plasma [23]. Their study did not show the existence of statistical significance between the groups of subjects 12 months after surgery (0.6 ± 0.9 vs. 1 ± 1.4). Comparing their results with the results of our research, it can be concluded that the values of their control group are almost identical to the values of the standard group (1 ± 1.4 vs. 0.9 ± 0.79). Comparison of VAS scores during the kneeling test indicated an advantage to the Vivostat group in our study compared with the PRP group of Cervelin’s research (0.35 ± 0.75 vs. 0.6 ± 0.9). Considering that the number of subjects in both studies was identical, in both groups, kneeling pain and donor site morbidity were evidence using VAS scoring; Vivostat is preferred in treatment since it yields a slightly better result compared with PRP.

Our study definitively showed that filling of the bone defect occurred earlier in the Vivostat group compared with the standard group. Pain in the anterior knee region primarily arises from the irritation of soft tissues and nerve endings along the edge of the defect itself, and kneeling transfers body weight to the front of the knee, so increasing the pressure on these tissues results in intensified pain. This study proved that the differences in bone defect filling and the pain intensity levels were noted at the second follow-up examination in both cases, but in favor of the Vivostat group.

Bioregenerative medicine is undoubtedly experiencing a huge expansion in recent decades, with very good and reproducible results. Using Vivostat^®^ PRF to fill a donor site defect is very simple, and the results of this study can be useful in clinical surgical practice, significantly contributing to the reduction in discomfort of the donor site region in surgically treated patients.

## 5. Conclusions

Anatomical reconstruction of the anterior cruciate ligament of the knee using a BTB graft is the most widely accepted technique globally, with better postoperative results compared with all the previously used operative techniques. Despite this, donor site pathology remains one of the biggest disadvantages of choosing this graft. Our study did show that the use of Vivostat^®^ PRF significantly contributes to the faster reduction in the width of the bone defect, as well as to the reduction in subjective complaints (the presence and intensity of pain during a kneeling test), but still does not solve the symptoms and limitations completely. This implies that further research into these problems, from the method of graft harvesting to the use of bioregenerative materials, as well as conducting more research on a larger number of patients with a longer follow-up period, may determine the most efficient treatment concept.

## Figures and Tables

**Table 1:** Patients’ characteristics.

Characteristics	Total	Vivostat	Standard	Test
Gender				
Male	40 (100%)	20 (100%)	20 (100%)	**-**
Age (years)				
Mean (SD)	27 (7.7)	26.2 (8.2)	27.8 (7.4)	ns *
Median (Range)	24.5 (17–44)	24 (17–42)	25.5 (19–44)	
BMI (kg/m^2^)				
Mean (SD)	25.9 (2.8)	25.7 (2.5)	26.1 (3.0)	ns *
Median (Range)	25.1 (21.6–35.4)	25.4 (21.6–31.2)	25.1 (22.9–35.4)	
Length of time ^#^ (months)				
Mean (SD)	14.8 (24.8)	18.5 (32.9)	11.2 (12.3)	ns *
Median (Range)	7.5 (0.4–144)	7 (0.4–144)	8 (1–42)	
Operated knee				
Right	15 (37.5%)	5 (25%)	10 (50%)	ns **
Left	25 (62.5%)	15 (75%)	10 (50%)	
Dominant leg injuries				
Right	19 (47.5%)	7 (35%)	12 (60%)	ns **
Left	21 (52.5%)	13 (65%)	8 (40%)	
**Total**	**40 (100%)**	**20 (100%)**	**20 (100%)**	**-**

ns—not statistically significant; ^#^ Length of time between injury and treatment; * Wilcoxon rank-sum test; ** Pearson chi-squared test.

**Table 2:** Bone defect depths measured by magnetic resonance imaging.

Depth of Bone Defect (mm)	Total	Vivostat	Standard	Test
Postoperative values				
Mean (SD)	6.7 (1.7)	6.7 (1.9)	6.7 (1.6)	ns *
Median (Range)	6.9 (3.3–12.4)	7.0 (3.3–12.4)	6.68 (4.5–10.0)	
4 months after surgery				
Mean (SD)	4.4 (1.4)	4.3 (1.3)	4.4 (1.4)	ns *
Median (Range)	4.5 (2.1–7.9)	4.5 (2.1–6.7)	4.5 (2.2–7.9)	
8 months after surgery				
Mean (SD)	3.4 (1.1)	3.3 (1.1)	3.4 (1.1)	ns *
Median (Range)	3.5 (1.4–6.0)	3.5 (1.4–6.0)	3.5 (1.7–5.6)	
12 months after surgery				
Mean (SD)	2.9 (0.9)	2.8 (1)	2.9 (0.8)	ns *
Median (Range)	3.0 (1.2–5.7)	3.0 (1.2–5.7)	2.9 (1.6–4.5)	
**Test between controls**				
All controls (Friedman Test)				
Postoperative vs. 4 ^#^ vs. 8 ^#^ vs. 12 ^#^	***p* < 0.01**	***p* < 0.01**	***p* < 0.01**	**-**
Pair of controls (Wilcox Signed-Rank Test)				
Initial vs. 4 ^#^	*p* ** < 0.0083	*p* ** < 0.0083	*p* ** < 0.0083	**-**
Initial vs. 8 ^#^				
Initial vs. 12 ^#^				
4 ^#^ vs. 8 ^#^				
4 ^#^ vs. 12 ^#^				
8 ^#^ vs. 12 ^#^				
**Total**	**40 (100%)**	**20 (100%)**	**20 (100%)**	**-**

ns—not statistically significant; * Wilcoxon rank-sum test; ^#^—months after surgery; ** Bonferroni correction for multiple testing (*p* < 0.05/6 = 0.0083), with *p*-value < 0.0083 for each pair.

**Table 3:** Bone defect width measured by magnetic resonance.

Width of Bone Defect (mm)	Total	Vivostat	Standard	Test
Preoperative values				
Mean (SD)	10.3 (2.0)	10.0 (2.0)	10.6 (2.0)	ns
Median (Range)	10.3 (4.1–13.9)	10.4 (5.5–13.6)	10.3 (4.1–13.9)	
4 months after surgery				
Mean (SD)	8.1 (2.0)	7.6 (2.0)	8.6 (1.8)	ns
Median (Range)	8.2 (2.5–11.8)	7.6 (2.5–11.8)	8.6 (3.8–11.7)	
8 months after surgery				
Mean (SD)	6.5 (1.8)	5.8 (1.8)	7.1 (1.5)	***p* < 0.05**
Median (Range)	6.6 (2.0–9.5)	5.4 (2.0–8.6)	7.2 (3.5–9.5)	
12 months after surgery				
Mean (SD)	5.2 (1.5)	4.6 (1.5)	5.8 (1.2)	***p* < 0.05**
Median (Range)	5.0 (1.2–7.9)	4.4 (1.2–7.4)	5.8 (3.3–7.9)	
**Test between controls**				
All controls (Friedman Test)				
Initial vs. 4 ^#^ vs. 8 ^#^ vs. 12 ^#^	***p* < 0.01**	***p* < 0.01**	***p* < 0.01**	**-**
Pair of controls (Wilcoxon Signed-Rank Test)				
Initial vs. 4 ^#^	*p* ** < 0.0083	*p* ** < 0.0083	*p* ** < 0.0083	**-**
Initial vs. 8 ^#^				
Initial vs. 12 ^#^				
4 ^#^ vs. 8 ^#^				
4 ^#^ vs. 12 ^#^				
8 ^#^ vs. 12 ^#^				
**Total**	**40 (100%)**	**20 (100%)**	**20 (100%)**	**-**

ns—not statistically significant; ^#^—months after surgery; ** Bonferroni correction for multiple testing (*p* < 0.05/6 = 0.0083), with *p*-value < 0.0083 for each pair.

**Table 4:** Preoperative and postoperative results of the subjective tests.

Type of Score	Total	Vivostat	Standard	Test *
**Modified Cincinatti score**				
Initial (preoperative) values				
Mean (SD)	65.5 (20.2)	68.3 (18.5)	62.7 (21.9)	ns
Median (Range)	65 (22–100)	68 (34–100)	63 (22–91)	
12 months after surgery				
Mean (SD)	93.1 (8.2)	92.4 (8.6)	93.8 (8.0)	ns
Median (Range)	96 (70–100)	94.5 (75–100)	96.8 (70–100)	
Wilcoxon Signed-Rank Test				
12 months vs. initial	***p* < 0.01**	***p* < 0.01**	***p* < 0.01**	-
**Activity score**				
Initial (preoperative) values				
Mean (SD)	6.8 (2.6)	6.8 (2.6)	6.7 (2.7)	ns
Median (Range)	7 (0–10)	7 (2–10)	7 (0–10)	
12 months after surgery				
Mean (SD)	6.6 (2.1)	6.6 (2.1)	6.6 (2.1)	ns
Median (Range)	6 (3–10)	6 (3–10)	6.5 (3–10)	
Wilcoxon Signed-Rank Test				
12 months vs. initial	ns	ns	ns	-
**IKDC score**				
Initial (preoperative) values				
Mean (SD)	57.8 (19.3)	61.3 (18.3)	54.2 (20.2)	ns
Median (Range)	58.0 (17.2–93.1)	64.4 (21.8–88.5)	55.2 (17.2–93.1)	
12 months after surgery				
Mean (SD)	89.9 (10.3)	89.2 (11.2)	90.6 (9.5)	ns
Median (Range)	90.8 (52.9–100)	90.8 (52.9–100)	91.4 (67.8–100)	
Wilcoxon Signed-Rank Test				
12 months vs. initial	***p* < 0.01**	***p* < 0.01**	***p* < 0.01**	-
**Tegner Lysholm score**				
Initial (preoperative) values				
Mean (SD)	71.2 (21.2)	74.8 (17.5)	67.4 (24.2)	ns
Median (Range)	77 (16–100)	79 (39–100)	76 (16–91)	
12 months after surgery				
Mean (SD)	93.5 (14.4)	95.6 (5.4)	91.4 (19.7)	ns
Median (Range)	95 (10–100)	97 (81–100)	95 (10–100)	
Wilcoxon Signed-Rank Test				
12 months vs. initial	***p* < 0.01**	***p* < 0.01**	***p* < 0.01**	-
**Total**	**40 (100%)**	**20 (100%)**	**20 (100%)**	**-**

ns—not statistically significant; * Wilcoxon rank-sum test.

**Table 5:** Correlations between bone defect dimensions (depth and width) and pain intensity (VAS) during the kneeling test (for all patients; *N* = 40).

Bone Defect Dimensions	Pain Intensity (VAS) during the Kneeling Test (after Surgery)		
	4 Months	8 Months	12 Months
**Depth of bone defect (mm)**			
Postoperative values			
Spearman’s rho	rho = −0.10	rho = −0.006	rho = 0.06
*p*-value	*p* = 0.52	*p* = 0.97	*p* = 0.70
4 months after surgery			
Spearman’s rho	rho = −0.07	rho = −0.01	rho = −0.08
*p*-value	*p* = 0.68	*p* = 0.94	*p* = 0.62
8 months after surgery			
Spearman’s rho	-	rho = −0.02	rho = −0.07
*p*-value		*p* = 0.88	*p* = 0.68
12 months after surgery			
Spearman’s rho	-	-	rho = −0.03
*p*-value			*p* = 0.87
**Width of bone defect (mm)**			
Postoperative values			
Spearman’s rho	rho = 0.15	rho = 0.23	rho = 0.17
*p*-value	*p* = 0.33	*p* = 0.15	*p* = 0.28
4 months after surgery			
Spearman’s rho	rho = 0.01	rho = 0.24	rho = 0.20
*p*-value	*p* = 0.94	*p* = 0.14	*p* = 0.21
8 months after surgery			
Spearman’s rho	-	rho = 0.18	rho = 0.24
*p*-value		*p* = 0.27	*p* = 0.14
12 months after surgery			
Spearman’s rho	-	-	rho = 0.17
*p*-value			*p* = 0.28

**Table 6:** Kneeling pain (VAS).

Kneeling Pain (VAS)	Total	Vivostat	Standard	Test *
4 months after surgery				
Mean (SD)	1.7 (1.49)	1.7 (1.53)	1.7 (1.49)	ns
Median (Range)	2 (0–5)	2 (0–5)	1.5 (0–4)	
8 months after surgery				
Mean (SD)	1.12 (1.14)	0.75 (1.07)	1.5 (1.1)	***p* < 0.05**
Median (Range)	1 (0–4)	0 (0–4)	1 (0–3)	
12 months after surgery				
Mean (SD)	0.62 (0.81)	0.35 (0.75)	0.9 (0.79)	***p* < 0.01**
Median (Range)	0 (0–3)	0 (0–3)	1 (0–3)	
**Test between controls**				
All controls (Friedman Test)				
4 ^#^ vs. 8 ^#^ vs. 12 ^#^	***p* < 0.01**	***p* < 0.01**	***p* < 0.01**	-
Pairs of controls (Wilcoxon Signed-Rank Test)				
4 ^#^ vs. 8 ^#^	*p* ** < 0.0167	*p* ** < 0.0167	ns **	-
4 ^#^ vs. 12 ^#^	*p* ** < 0.0167	*p* ** < 0.0167	*p* ** < 0.0167	-
8 ^#^ vs. 12 ^#^	*p* ** < 0.0167	*p* ** < 0.0167	*p* ** < 0.0167	-
**Total**	**40 (100%)**	**20 (100%)**	**20 (100%)**	**-**

ns—not statistically significant; ^#^—months after surgery; * Wilcoxon rank-sum test; ** Bonferroni correction for multiple testing: *p* < 0.05/3 = 0.0167.

## Data Availability

All data used in this study can be obtained via a request to the following email: darkomil@doctor.com.
